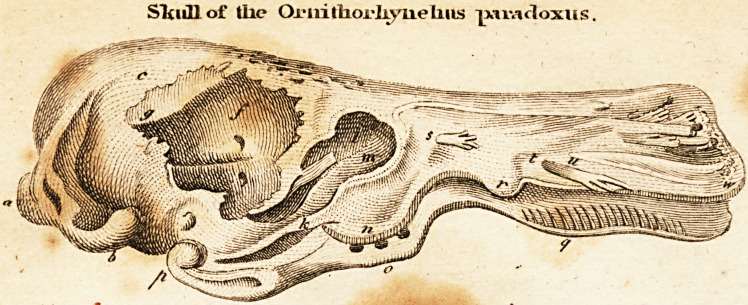# Some Anatomical Remarks on the Ornithorhynchus Paradoxus, from New South Wales

**Published:** 1801-07

**Authors:** 


Skull of the Ornithorhynchus paradoxus.

Hemispherical acoustic Tube. (No. 2.)

Medical Journal No. 29.

The Metallic Brush.

Printed for R. Phillips No. 71. St. Paul's Church Yard.

### *Some Anatomical Remarks on the* Ornithorhynchus Paradoxus, *from New South Wales; by Prof.* BLUMENBACH.

( With an Engraving. )

THE moſt remarkable circumſtance in this curious animal is the great ſimilarity of its head with that of a duck, which, however, is ſtill more ſtriking in its internal ſtructure. From the external form of the ſkull of this animal, one might be more eaſily led to conclude that it belonged to ſuch an aquatic bird, than to a creature of the Mammalia tribe. Both the jaws are as broad and low as in ducks, and the calvaria has, at leaſt in my ſpecimen, no traces of a future, as is generally the caſe in full-grown birds. There is likewiſe a ſingularity in the cavity of the ſkull, (caverna encephali) of which nothing like it is known in any quadruped animal of the Mammalia, though there exiſts ſomething analogous in the claſs of birds, viz. a conſiderable bony falx (g), which is ſituated along the middle of the *os frontis*, and the *oſſa bregmatis*. This proceſſus is in general ſcarcely to be ſeen in the Mammalia, even in thoſe that have a bony tentorium cerebelli. I know no inſtance of any animal of that tribe being provided with ſuch a bony falx as the Ornithorhynchus, (*schnabelthier*, as Pr. Bl. calls it, i. e. beak animal, or beaked animal) if we except ſome anomalous formations; as I poſſeſs the ſkull of a woman, in which the tabula vitrea of the os frontis has formed a ſimilar bony criſta. Amongſt the bird tribe, however, in which Willis and others deny the exiſtence of ſuch a criſta, I have diſcovered it in the ſkull of the cock of the wood, (urogallus), which ſurpriſingly agrees as well in the ſituation as in the form with that in the Ornithorhynchus. The mandible of this animal is very ſingular, conſiſting of a beak, the under part of which has its margin indented as in ducks, (q) and of the proper inſtrument for chewing that is ſituated behind, within the cheeks. This has, at leaſt in my ſpecimen, no teeth, nor even the traces of alveoli, but only two broad proceſſus of a peculiar formation on each ſide, whoſe undulated ſuperficies fit one another. (n. o.) Dr. SHAW ſays of the ſpecimen he examined, that it had no teeth, "dentium nulla ſunt veſtigia." Vid. No. 118 *of the Naturaliſt's Miſcellany*. But Sir JOSEPH BANKS, to whoſe liberality I am indebted for my ſpecimen, informs me, that Mr. Home, has found in a ſpecimen that belongs to the Society of Natural Hiſtory at Newcaſtle, on each ſide of the jaws, two ſmall and flat molar-teeth. The fore part of this anomalous mandible, or the beak, is covered and bordered with a coriaceous ſkin, in which three parts are to be diſtinguiſhed: 1. The proper integument of the beak, (integumentum roſtri). 2. The labiated margins of it, (margines labiales). 3. A curious edge of the ſkin of the beak, (limbus tranſverſarius). Into theſe three parts of that membrane numerous nerves are diſtributed, of which thoſe in the upper part of the beak ariſe from the ſecond branch of the fifth pair, viz. in the limbus tranſverſarius; that which penetrates through the foramen infraorbitale, (s) in the margo labialis; that which comes forth behind the oſſa intermaxillaria (t) and to the integumentum roſtri, three branches, which run out between the oſſa intermaxillaria, (u). From this quantity of nerves, with which the integument of the beak is provided, no doubt is left of this part being intended as the organ of feeling, a ſenſe which, beſides men and the quadrumanes, very few mammalia enjoy, that is to ſay, few animals poſſeſs the faculty of diſtinguiſhing the form of external objects and their qualities by organs deſtined for that purpoſe; a property that is different from the common feeling, by which every animal is able to perceive the temperature and preſence of ſenſible objects, but without being informed by the touch of them, of their peculiar qualities. Thus, for inſtance, the ſkin in the wings of a bat, and its ear, ſerve probably as organs of common feeling, by means of which they are enabled to flutter, even after being blinded, without flying againſt any thing. The whiſkers (vibriſae) of many animals ſeem likewiſe to ſerve for the purpoſe of informing them of the preſence of ſenſible bodies, on which account Dr. DARWIN compares them with the antennae of inſects; but they are not able to inform themſelves of the properties of thoſe objects. It is true, that the ſnout of a mole has been conſidered by DERHAM, and the ſnout and tongue of many other animals likewiſe by BUFFON, as organs of touching; but this ſeems only to be their ſecondary uſe. The ſame may be ſaid of the elephant's trunk, which BUFFON alſo conceives to be an organ of touching, although from its manner of living, the neceſſity of ſuch an organ of touching does not appear. The ornithorhynchus, however, is an animal, which from the ſimilarity of its abode, and the manner of ſearching for food, agrees much with a duck, on which account it has been equally provided by Nature with an organ for touching, viz. with the integument of the beak, richly endowed with nerves. This inſtance of analogy in the ſtructure of a ſingular organ of ſenſe in two ſpecies of animals from claſſes quite different, is highly inſtructive for comparative phyſiology, and on this account the ornithorhynchus belongs to one of the moſt remarkable markable phenomena in Zoology, and may in general be looked upon as one of the moſt intereſting diſcoveries with which that part of natural hiſtory has been enriched during the laſt century.

## Figures and Tables

**Figure f1:**